# Circulating microRNAs as potential biomarkers of physical activity in geriatric patients with HCV

**DOI:** 10.1186/s12860-024-00514-8

**Published:** 2024-07-19

**Authors:** Hadeel A. Al-Rawaf, Sami A. Gabr, Amir Iqbal, Ahmad H. Alghadir

**Affiliations:** 1 Department of Clinical Laboratory Sciences, College of Applied Medical Sciences, King Saud University, Riyadh, 11433 Saudi Arabia; 2 Department of Rehabilitation Sciences, College of Applied Medical Sciences, King Saud University, P.O. Box 10219, Riyadh, 11433 Saudi Arabia

**Keywords:** MicroRNAs, Pain, HCV, Physical activity, Antioxidant status, Muscle capacity

## Abstract

**Background:**

Circulating microRNAs have been implicated in a diverse array of biological and pathological phenomena. Their potential utility as noninvasive biomarkers for screening and diagnosing various diseases has been proposed.

**Objective:**

This study aimed to explore the potential role of the miRNAs miR-122 and miR-486 as molecular biomarkers in the pathogenesis of hepatitis C virus (HCV) infection. Thus, miR-122 and miR-486 were detected in the serum of HCV patients and healthy controls. Moreover, the potential correlations of miR-122 and miR-486 with viral complications, such as physical activity, pain, muscle fatigue, and HCV infection, were identified.

**Methods:**

A total of 150 subjects aged 30 to 66 years were included in this study. The patients were classified as patients with chronic hepatitis C virus (CHC) (*n* = 110) or healthy controls (*n* = 40). Real-time polymerase chain reaction (PCR) analyses were performed to determine miR-122 and miR-486 expression. Physical activity (PA), pain score, HCV genotyping, viral overload, aspartate transaminase (AST), alanine transaminase (ALT), lactic acid dehydrogenase (LDH), creatine kinase (CK), and antioxidant status were also estimated by using prevalidated questionnaires, PCR, and spectrophotometric analyses.

**Results:**

Compared with those in normal controls, significant increases in the serum levels of miR-122 and miR-486 were reported in patients with CHC. In physically active CHC patients, there was a significant correlation between the expression of miRNAs and increased alanine transaminase (ALT), aspartate transaminase (AST), fibrosis scores, and inflammation activity, but no association was reported for hepatitis C virus (HCV) RNA or viral load. Additionally, significant decreases in LDH, CK, GSSG, and pain scores and increases in TAC, GSH, and the GSH/GSSG ratio were reported. Moreover, the expression of miR-122 and miR-486 was positively correlated with changes in body mass index (BMI) and liver fibrosis stage, as well as negatively correlated with sex, PA, TAC, GSH, GSSG, and the GSH/GSSG ratio.

**Conclusion:**

MiR-122 and miR-486 expression levels were strongly correlated with physical activity, pain perception, and muscle fatigue biomarkers in HCV-infected patients. These miRNA levels were associated with elevated AST, ALT, fibrosis scores, LDH, CK, and antioxidant status, thus suggesting their potential as biomarkers for disease severity and oxidative stress. However, no correlation was observed with viral load or HCV-RNA expression, thus implying that these miRNAs may impact disease progression and symptoms through host factors, rather than directly affecting viral replication. In summary, the results demonstrated that molecular studies of miR-22 and miR-468 and their associations with PA, pain, adiposity, sex differences, and muscle fatigue, as well as routine biomarkers, could be useful as prognostic nanoninvasive biomarkers, thus providing novel therapeutic targets for CHC infection.

## Introduction

Chronic liver conditions, including liver cirrhosis, hepatic failure, and hepatocellular carcinoma, have been linked to chronic hepatitis C virus (HCV) infections in an estimated 170 million individuals worldwide [1, 2]. The progression of infection subsequently leads to severe liver complications, which can necessitate transplantation in many patients [3]. Thus, eradication of HCV infection significantly reduces liver-related morbidity and mortality [4–6].

HCV infection has its most pronounced impact on patients’ social and physical functioning, overall well-being, and vitality [7]. Current medical advice recommends the management and correction of health-related behaviors, including nutritional or physical activity, during viral infection to prevent further liver disease progression. Importantly, this refers to the influence of lifestyle on disease development [8–10].

Patients who are afflicted with HCV infection have reported experiencing diminished self-assurance and a decrease in their physical capabilities [11, 12]. This decrease in quality of life, which is linked to chronic hepatitis, may be influenced by both comorbid conditions and the patient’s awareness of their diagnosis rather than being solely dependent on the severity of the viral infection, liver function, or the extent of fibrosis [7, 13].

The advantages of physical exercise are multifaceted and extend to mitigating risks in individuals with various chronic conditions [14–17]. Indeed, physical exercise can play a crucial role in the management of chronic diseases [18]. Earlier studies have suggested the incorporation of physical activity as an integral component in supporting individuals who are experiencing chronic HCV infection, with an aim of bolstering their self-assurance and overall capabilities [19, 20]. In physically active HCV patients, their bodies manufacture ATP more often, thus indirectly boosting the body’s glutathione levels.

This scenario correspondingly improves the resilience of the liver against free radicals and hepatitis C damage [21]. Using the aforementioned data, we can determine that there is no direct correlation between viral infection, disease progression, or physiological changes; moreover, only a correlation with antioxidant activity has been reported in patients with HCV [7, 13, 18, 21].

Thus, in our hypothesis, we suggest that there is a relationship between physical activity and HCV infection, which may be clarified by investigating other biomarkers, such as microRNAs (miRNAs). MicroRNAs, which are short noncoding RNAs consisting of approximately 22 nucleotides, are involved in posttranscriptional gene expression regulation. They have been demonstrated to participate in a broad spectrum of biological and pathological processes [22–24].

Recently, there has been growing interest in assessing the potential of miRNAs as being noninvasive biomarkers for the screening and diagnosis of various diseases. The ability of circulating miRNAs to serve this purpose may be attributed to their presence in exosomes, which provide stability [25–28]. In the human genome, over 2,500 miRNAs have been identified, and they are likely involved in a wide range of cellular processes, including (but not limited to) development, differentiation, proliferation, apoptosis, disease pathology, and antiviral defense [29, 30].

In the context of HCV infection, several miRNAs are significantly upregulated in different HCV genotypes, with notable examples being serum miR-134, miR-198, miR-320c, and miR-483-5p, which show promise as being diagnostic biomarkers for HCV infection [31]. Among all human miRNAs, microRNA 122 (miR-122) is one of the most highly expressed and abundant miRNAs, constituting more than 70% of the total miRNA content in the human liver. Consequently, it plays a pivotal role in the regulation of hepatic functions [32]. Moreover, research has indicated that miR-122 is indispensable for HCV replication and may serve as a target for anti-HCV therapy [33]. Additionally, there is a body of evidence linking physical activity to skeletal muscle development [34–36], and miRNAs are believed to act as physiological mediators of the adaptations induced by exercise [37]. Thus, miRNAs represent interesting factors in the study of host-HCV interactions, ranging from HCV infection and related consequences such as low physical activity, fatigue, and depression to potential novel targets for antiviral therapy [38, 39].

Additionally, several studies have shown that the molecular signatures of cellular miRNA expression may significantly control the classification, diagnosis, and prognosis of cancer [40, 41]. However, the diagnostic and prognostic accuracy of the miRNA genetic fingerprint for more than 16,000 mRNAs were significantly different in different cancers, including the incidence rates of hepatocellular carcinoma among patients at high risk of hepatitis C virus infection [41, 42]. The biological functions and targets of several miRNAs, such as miR-618 and miR-650, have been reported as being noninvasive diagnostic markers [42] that can be identified in urine samples and are valuable approaches for developing screening methods that can reduce mortality rates among HCV-infected patients [43–47].

In addition, several studies have shown that cellular miR-486 is a multifunctional miRNA involved in many cancers, including the tumorigenesis of hepatocellular carcinoma, with additional functional roles as a modulator for regulating drug resistance, such as sorafenib resistance, by targeting FGFR4 and EGFR, thus offering a potential target for HCC treatment [44–47]. Although the potential roles of both miR-22 and miR-486 as prognostic markers in the diagnosis, treatment, and prevention of drug resistance have been addressed in many studies [40–47], little is known about their association with physical activity, pain, and muscle fatigue as complications in patients with HCV infection. Thus, this study aimed to elucidate the potential role of miR-122 and miR-486 in relation to physical activity, pain, and muscle fatigue biomarkers in patients with HCV infection or its complications, as well as to provide valuable insights into the differences between patients with HCV infection and healthy individuals.

## Materials and methods

### Study design

This study employed a case‒control observational design to determine the relationships between miRNA-122 and miR-486 expression and physical activity, pain, and muscle fatigue biomarker levels in patients with HCV infection or its complications, as well as to compare these findings with those observed in the healthy control group.

### Ethical considerations

The study protocol underwent review and received approval from the ethics subcommittee at King Saud University, Riyadh, Saudi Arabia, with the assigned file number ID: RRC-2016-045. The research adhered to ethical principles outlined by the local institutional review board and was conducted in accordance with the Declaration of Helsinki (2010). Prior to the commencement of the study, all of the participants were provided with comprehensive information about the research, and each participant provided their informed consent through a written and signed document.

### Subjects

A total of 150 older adults, ranging in age from 30 to 66 years, met the eligibility criteria for participation in this study. The study included a control group consisting of 40 healthy HCV-negative individuals who exhibited normal levels of physical activity (with a PA score ≥ 2,500 MET minutes/week). Additionally, a total of 110 patients with chronic HCV infection, as well as with a confirmed disease duration of more than 10 years, viremia, HCV antibodies as indicated by the RIBA-II test, elevated serum transaminase levels surpassing the upper normal limit, HCV-RNA positivity, and genotype determinations, were included in the current study. The exclusion criteria included individuals with viral coinfections (HIV and/or HBV), advanced stages of other liver diseases (such as cirrhosis), hepatocellular carcinoma, prior liver transplantation, obesity (BMI ≥ 25 and ≥ 30 kg/m^2^), iron overload, or insufficient liver biopsy results. Heparinized syringes were utilized to collect blood samples from all of the subjects either on the day of the biopsy or within 5 days following the biopsy procedure. Subsequently, serum samples were extracted through centrifugation, which lasted for 1 min at 1,400 rpm. These samples were assigned coded study identification numbers and were then promptly frozen at -20 °C for subsequent analysis. A comprehensive summary of the participants’ demographic and clinical information can be found in Table 1.

**Table 1:** Clinical and demographics characteristics of control and geriatric CHC patients

Parameters	CHC patients (*n* = 110)	Healthy controls (*n* = 40)
Age (years) ^#^	46.5 ± 5.8	46.9 ± 5.1
Sex (male/female) ^#^	75/35	25/15
BMI (kg/m2) ^#^	24.3 ± 4.6^*^	22.8 ± 6.3
AST (IU/mL) ^# #^	69.4 ± 9.5^**^	18.3 ± 3.5
ALT (IU/mL) ^# #^	115.4 ± 21.5^**^	23.4 ± 5.8
Albumin (gm/dL) ^#^	2.8 ± 0.89^**^	4.3 ± 0.75
Bilirubin (mg/dL) ^#^	3.9 ± 0.96^**^	0.81 ± 0.65
AFP (ng/mL)	38.1 ± 3.6	2.8 ± 3.1
Platelets (109/L) ^#^	175 ± 25.4^**^	254 ± 34.7
Glucose (mg/dL)	91.7 ± 2.5^*^	82.5 ± 3.9
HOMA-IR	4.25 ± 1.6^*^	1.7 ± 0.53
Duration of HCV (years)	11.5 ± 3.1	-
HCV RNA (IU/mL)	9.5 × 10^5^ ±2.25 × 10 ^5^	-
HCV genotypes	G 4	-
Viral load	441.8 ± 3.5	-
Length of liver-biopsy core	22.5 ± 0.75 cm	-
Portal tracts	16 ± 3.8	-
ASC/fibrosis/cirrhosis	48/35/27	-
Necroinflammation; n (%) A0–A1 A2–A3	45 (40.9%) 65 (59.1%)	- -

**Notes**: All values represent mean ± standard deviation or n (%). These parameters are related to viral estimation and hepatic pathology; ^#^Student’s t-test; ^##^Mann–Whitney U-test; *P < 0.05; **P < 0.01. **Abbreviations**: CHC, chronic hepatitis C; BMI, body mass index; HCV, hepatitis C virus; HOMA, homeostatic model assessment; IR, insulin resistance; ASC, asymptomatic CHC; AST, Aspartate aminotransferase; ALT, Alanine aminotransferase

### Laboratory assessment

The levels of liver enzymes, such as alanine aminotransferase (ALT), aspartate aminotransferase (AST), albumin (ALB), and total bilirubin (TB), were measured in the sera of both healthy controls and HCV-infected patients. In addition, complete blood platelet count, glucose, and HOMA-IR were also estimated according to the manufacturer’s instructions. Serum AFP levels were measured via sandwich enzyme-linked immunosorbent assay (ELISA; R&D Systems, Minneapolis, MN, USA).

### Assessment of HCV infection

As a measure of diagnosis, HCV-RNA was also estimated qualitatively by using a nested PCR Qiagen RNA-extraction kit (Thermo Fisher Scientific, Waltham, MA, USA) and quantitatively by using Smart Cycler II real-time PCR (Cepheid, Sunnyvale, CA, USA) with HCV RNA quantification kits (Sacace Biotechnologies, Como, Italy) for estimation of HCV RNA-positive subjects, as previously described [48, 49]. Moreover, subjects with an HCV viral load lower than 250 IU/ml were considered negative. In addition, reverse hybridization was achieved to estimate the HCV genotype by using a line-probe assay (Inno-LiPA HCV II kit; Innogenetics, Ghent, Belgium) [50].

In this regard, patients with alanine aminotransferase (ALT) levels higher than the upper limit of normal, elevated titers of anti-HCV antibodies estimated with a third-generation enzyme immunoassay (AxSym HCV 3.0; Abbott Laboratories, Abbott Park, IL, USA), and confirmed levels of HCV-RNA by PCR analysis were diagnosed with chronic hepatitis C (CHC).

### Histologic examination

The degree of liver inflammation in CHC patients was assessed through the use of the Batts and Ludwig scoring system [51]. After informed consent documents had been submitted, hepatic biopsies were obtained from all of the patients by a surgeon after computed tomography or magnetic resonance imaging. The histological diagnosis of cirrhosis and HCC was based on an elevated serum AFP level (≥ 400 ng/mL) and computed tomography or magnetic resonance imaging scans, as recommended by international criteria. Thus, a preoperative clinical diagnosis of primary liver cancer and hepatocellular carcinoma (HCC) was made as described previously [52, 53].

For histological examination, liver biopsies were obtained by using an automatic 16-gauge Trucut needle (biopsy gun), which provides adequate specimens for evaluation and fewer cases with tissue fragmentation. The analyzed liver biopsy specimens were at least 15–25 mm long with complete portal tracts (> 10). Formalin-fixed, paraffin-embedded sections were stained with hematoxylin and eosin and Masson’s trichrome. Slides were labeled with patient identification numbers and subsequently reviewed and graded blindly by a senior pathologist. The mean length of liver biopsies and the number of portal tracts were assessed (including only the complete, intact portal tracts) [54–56].

The degree of fibrosis was scored according to the Metavir system, and no fibrosis was defined as F0, mild fibrosis as F1, moderate fibrosis as F2, severe fibrosis as F3, and cirrhosis as F4. Significant fibrosis was also defined as F2–F4. Hepatic inflammatory activity was also scored [54, 55].

### Isolation of miRNAs and RT–PCR

Total RNA from the serum samples of each subject was extracted by using TRIzol LS reagent (Invitrogen, Carlsbad, CA) and subsequently subjected to reverse transcriptase PCR (RT‒PCR) analysis. A ready-made solution containing primers and probes for human miR-122 and miR-486 (Applied Biosystems, Foster City, CA) and real-time RT–PCR was generated by using an ABI 7300 system (Applied Biosystems) [46]. After the addition of phenol solution to inhibit RNase activity, chemically synthesized cel-miR-39 (Sigma‒Aldrich Japan, Tokyo, Japan) was added as an exogenous and endogenous control, and miR-16, which is a representative miRNA enriched in blood, was added as an endogenous control. For each serum sample, the ratio of the miRNA signal to that of endogenous (miR-16) and exogenous (cel-miR-39) controls was calculated as previously reported [57].

### Serum biochemical analyses

A standard commercially available enzymatic assay (Granutest 15, EMD Millipore, Billerica, MA, USA) was used to calculate the serum creatine kinase (CK) concentration. Lactate dehydrogenase (LDH) activity was estimated by using an ultraviolet method provided by Randox Laboratories Ltd. (Antrim, UK). Serum alanine transaminase (ALT), aspartate transaminase (AST), albumin, bilirubin, blood platelet count, and fasting blood glucose were estimated in serum and blood samples by using standard methods. The serum total antioxidant capacity (TAC) was calculated by using a colorimetric assay kit (K274-100; BioVision, Milpitas, CA, USA). The data were measured and calculated as previously reported [58, 59]. The serum samples were utilized for spectrophotometric assays of glutathione (GSH) and glutathione disulfide (GSSG) levels, which was conducted by employing an enzyme-linked immunosorbent assay (ELISA) reader (Tecan GENios, A-5082, Tecan, Salzburg, Austria) and a glutathione assay kit (Cayman Chemical Company, Ann Arbor, MI, USA). The concentrations of GSH and GSSG were determined at 405 nm based on the color development of the TNB (5-thio-2-nitrobenzoic acid) product resulting from the reaction between GSH and DTNB (5,5’-dithio-bis-2-nitrobenzoic acid) [60].

### Assessment of physical activity (PA)

A prevalidated global PA questionnaire (GPAQ) was used to calculate the PA scores of all participants regarding the intensity of exercise performed in minutes or hours per day for each participant, as previously reported [61, 62]. In relation to the intensity and frequency of PA achieved per week, energy expenditure was measured in the form of the metabolic equivalent (MET) of all of the participants. Thus, according to energy expenditure, PA scores were classified into three scores: physically inactive (MET minutes/week of ≤ 500), moderate PA (MET minutes/week of 500–2,500), and physically active (≥ 2,500 MET minutes/week) [61, 62]. Using the PA scores as a criterion, HCV patients were divided into two categories: those who were physically active (≥ 2,500 MET minutes/week, *n* = 85) and those who were PA inactive (MET minutes/week of ≤ 500, *n* = 25).

### Assessment of VAS score

A reliable standard 100 mm visual analog scale (VAS), with 0 mm indicating “no pain” and 100 mm indicating “most severe pain”, was used to measure the pain intensity of all of the participants, as previously reported [63, 64].

### Statistical analyses

The Shapiro–Wilk test was used to assess the normality of the distribution of the data, and logarithmic transformation was applied for subsequent statistical analyses. To evaluate differences between subject groups, both Student’s t test and ANOVA analyses were utilized, followed by Bonferroni’s multiple comparison analysis. To compare microRNA levels among different groups, adjustments were made using univariate analysis through a general linear model. Multiple stepwise regression and Pearson’s correlation analysis were employed to determine the relationships between microRNA levels, TAC, LDH, CK, GSH/GSSG, HCV, and metabolic parameters.

## Results

A study involving 150 participants was conducted, with the majority being male (66.66%). The clinical and demographic characteristics of both the control group and the CHC patients are presented in Table 1. CHC patients exhibited significantly greater values (*P* = 0.001) in terms of BMI, AST, ALT, platelet count, and glucose status than did control subjects.

In this study, LDH, CK, TAC, GSH, GSSG, and the GSH/GSSG ratio were measured as predictors of muscle function capacity and physical activity in controls and patients with CHC (Table 2). In physically active CHC patients, LDH, CK, and GSSG were significantly lower, whereas TAC, GSH, and the GSH/GSSG ratio were significantly greater than those in CHC patients with lower PA scores (*P* = 0.001) or in the control group (*P* = 0.01). Additionally, pain was significantly greater in patients with CHC who had lower PA scores than in both controls (*p* = 0.01) and PA patients with CHC (*P* = 0.001).

**Table 2:** Change in muscle and antioxidant biomarkers, and pain scores (VAS) of control and geriatric CHC patients based up on PA (Mean ± SD).

Parameters	PA control (*n* = 40)	CHC patients (*n* = 110)	
		PA-Active, *n* = 85 (≥ 2,500 MET min/week)	PA-Inactive, *n* = 25 (≤ 500 MET min/week)
LDH(IU/L)	18.9 ± 3.1	29.5 ± 6.4^a^	48.6 ± 9.1 ^a, b^
CK (UI/L)	168.6 ± 8.4	156.7 ± 5.9 ^a^	210.2 ± 5.3 ^a, b^
TAC (nmol/µL)	45.8 ± 12.3	36.4 ± 6.7 ^a^	18.1 ± 3.8 ^a, b^
GSH (µmol/L)	1320 ± 412	815 ± 251 ^a^	521 ± 125 ^a, b^
GSSG (µmol/L)	86.5 ± 22.8	135.7 ± 51.2 ^a^	215 ± 65.9 ^a, b^
GSH/GSSG ratio(×10 ^− 2^)	26.3 ± 11.4	9.5 ± 4.5 ^a^	4.6 ± 2.6 ^a, b^
VAS score	12.3 ± 2.5	16.7 ± 4.7 ^a^	48.2 ± 12.4 ^a, b^

**Notes**: Values are expressed as mean ± SD; ^a^*P*<0.01 (CHC vs. control), ^b^*P*<0.001 (PA-inactive vs. PA active). Significance at *P* < 0.05. **Abbreviations**: CK, creatine kinase; LDH, lactate dehydrogenase; MET, metabolic equivalent; PA, physical activity; SD, standard deviation; TAC, total antioxidant capacity; VAS, visual analog scale; GSH, reduced glutathione; GSSG, oxidized glutathione

**Fig. 1:**
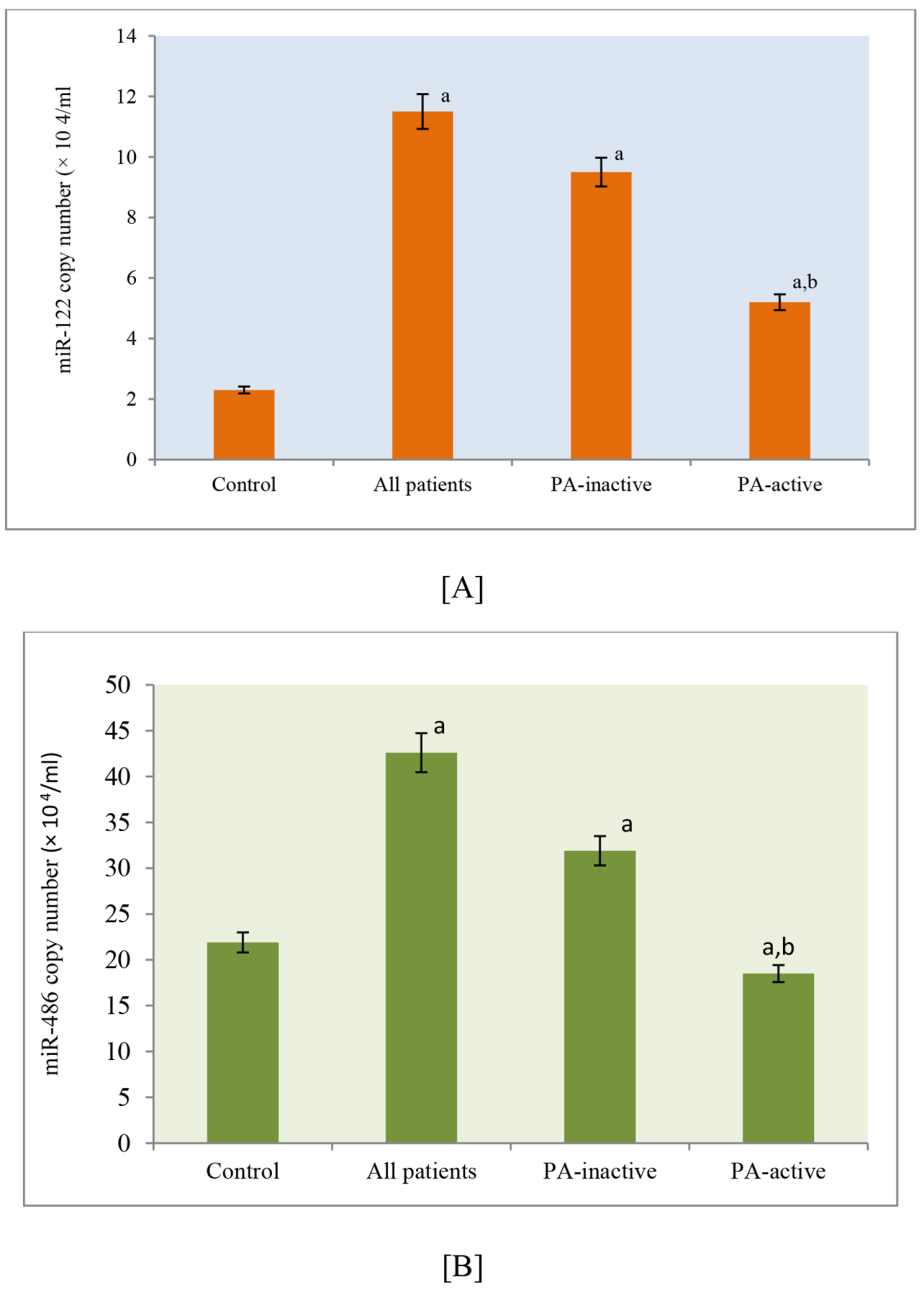
Elevation in serum miR-122 and miR-486 expression in both physically active and non-active chronic hepatitis C. Serum levels of miR-122 (**A**) and miR-486 (**B**) were compared between control subjects and CHC patients. The CT values of serum miRNA expression levels were transformed into absolute values using standard curves. The bars represent the levels corresponding to CT values ranging from 10 to 12 (2.9 × 104 − 0.75 × 104 copies/ml) for miR-122 and 40 to 45 (1.75 × 104 − 0.68 × 104 copies/ml) for miR-486, respectively

To explore the expression levels of miRNAs and their potential associations with physical activity patterns in CHC patients, we examined whether miR-122 and miR-486 were detectable and modulated in the serum of CHC patients. The serum concentrations of miR-122 and miR-486 were notably greater in all of the CHC patients (*n* = 110) than in the HCs (*n* = 40) (miR-122: 11.5-fold vs. 2.3-fold, respectively; *P* = 0.0001; miR-486: 42.6-fold vs. 21.9-fold, respectively; *P* = 0.0001) (Fig. 1A-B).

Among CHC patients who engaged in regular physical activity, the serum expression levels of both miR-122 and miR-486 were significantly lower than those in CHC patients with lower physical activity levels (miR-122: 5.2-fold vs. 9.5-fold, respectively; *p* = 0.0001; miR-486: 18.5-fold vs. 31.9-fold, respectively; *p* = 0.0001) (Fig. 1A-B).

The correlations of miR-122 and miR-486 with demographic, physical activity, muscle capacity, antioxidant, and viral parameters were further studied by using stepwise regression and Pearson’s correlation analyses (Table 3). Both miR-122 and miR-486 were positively correlated with glucose levels (*P* = 0.01) and BMI (*p* = 0.001) and negatively correlated with sex (*p* = 0.002).

**Table 3:** Stepwise regression analyses and Pearson’s correlation of miR-122 and miR-486 with indices of HCV infectivity and muscle functional capacity in CHC patients

Covariables	Pearson’s correlation				Stepwise			
	miR-122		miR-486		miR-122		miR-486	
	*r*	*P*-value	*r*	*P*-value	β	*P*-value	β	*P*-value
Age	0.032	0.365	0.124	0.312	0.045	0.524	0.031	0.325
Sex	-0.156	0.002	-0.163	0.002	-0.054	0.317	-0.042	0.128
BMI	0.325	0.001	0.325	0.001	0.037	0.112	0.025	0.613
HCV RNA	0.215	0.214	0.152	0.145	0.058	0.614	0.081	0.215
HCV viral load	0.298	0.568	0.325	0.665	0.075	0.234	0.081	0.128
ALT (IU/L)	0.98	0.001	0.89	0.001	0.051	0.01	0.061	0.01
AST(IU/L)	0.78	0.01	0.92	0.01	0.042	0.05	0.075	0.05
Fibrosis score	0.112	0.001	0.311	0.001	0.045	0.001	0.084	0.001
Inflammation activity	0.58	0.001	0.61	0.001	0.031	0.02	0.054	0.02
Glucose (mg/dl)	0.35	0.01	0.39	0.01	0.28	0.01	0.36	0.02
LDH	0.258	0.001	0.368	0.001	0.025	0.001	0.057	0.001
CK	0.35	0.001	0.39	0.001	0.054	0.01	0.012	0.01
TAC	-0.25	0.001	-0.035	0.001	-0.043	0.001	-0.058	0.001
GSH	-0.45	0.001	-0.054	0.001	-0.036	0.001	-0.065	0.001
GSSG	-0.78	0.01	-0.084	0.01	-0.075	0.01	-0.095	0.01
GSH/GSSG ratio	-0.34	0.01	-0.042	0.01	-0.051	0.01	-0.074	0.01
PA-scores	-0.48	0.05	-0.056	0.05	-0.032	0.01	-0.071	0.01
VAS score	0.96	0.001	0.085	0.001	0.074	0.001	0.068	0.001

r, Pearson’s correlation coefficient; β, standardised regression coefficient; ‘–’, indicates that the variable was not included in the multiple stepwise regression modelling. **Abbreviations**: CK, creatine kinase; LDH, lactate dehydrogenase; MET, metabolic equivalent; PA, physical activity; SD, standard deviation; TAC, total antioxidant capacity; VAS, visual analog scale; GSH, reduced glutathione; GSSG, oxidized glutathione

Furthermore, we assessed the activities of ALT and AST in CHC patients and observed a significant increase compared to those in control subjects. Additionally, we found that the relative expression of both miRNAs (miR-122 and miR-486) exhibited a moderately positive correlation with the serum ALT and AST levels (miR-122: *r* = 0.98, *P* = 0.001 and *r* = 0.78, *P* = 0.01, respectively; miR-486: *r* = 0.89, *P* = 0.001 and *r* = 0.92, *P* = 0.01, respectively) (Table 3). However, no correlation was detected between the expression levels of these miRNAs and either HCV-RNA (*r* = 0.215; *P* = 0.21) or the viral load (*r* = 0.298, *P* = 0.56) in CHC patients. Notably, both miR-122 and miR-486 exhibited positive correlations with liver fibrosis score (*P* = 0.001) and inflammatory activity (*P* = 0.001) in both physically active and nonactive CHC patients.

Regarding physical activity and muscle capacity, the expression of miRNAs in CHC patients was positively correlated with LDH and CK and negatively correlated with PA score, TAC, GSH, GSSG, and the GSH/GSSG ratio (Table 3). In addition, pain intensity was significantly correlated with the levels of both miR-122 (*P* = 0.001) and miR-486 (*P* = 0.001).

**Fig. 2:**
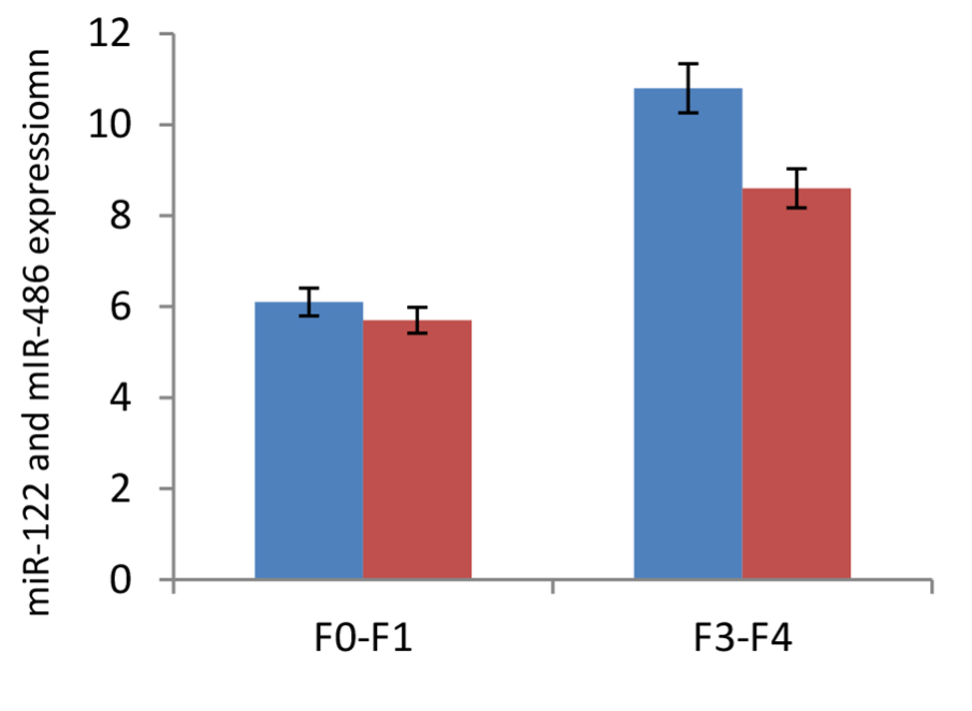
Expression levels of miR-122 and miR-486 in serum of CHC patients with early (F0-F1) and advanced (F3-F4) liver fibrosis. Serum levels of miR-122 and miR-486 were elevated in patients with advanced liver fibrosis (miR-122, 10.8-fold; miR-486, 8.6-fold) compared to those with early liver fibrosis scores (miR-122, 6.1-fold; miR-486, 5.7-fold)

Based on histological grading, the patients were categorized into two groups: those with an early stage of fibrosis (F0-F1) and those with advanced fibrosis (F3-F4). We found that the serum levels of miR-122 and miR-486 were significantly greater (*P* = 0.001) in patients with advanced liver fibrosis (miR-122, 10.8-fold; miR-486, 8.6-fold) than in those with early liver fibrosis (miR-122, 6.1-fold; miR-486, 5.7-fold) (Fig. 2). Notably, the increase in miR-122 expression was predominantly observed in patients with F2-F4 disease, whereas the increase in miR-486 expression was consistent with that in patients with F0-F3 disease and further elevated in patients with F4 disease. In addition, lower expression of the two miRNAs was reported in the livers of physically active patients than in those of patients with lower PA or sedentary lifestyles at comparable fibrosis stages (Table 4).

**Table 4:** Correlation between serum levels of miR-122 and miR-486 and histological liver severity in CHC patients

	Liver fibrosis scores				
CHC patients	F0	F1	F2	F3	F4
PA-patients					
miR-122 miR486	2.8 × 10^4^±0.65 × 10^4^ 1.3 × 10^4^±0.35 × 10^4^	3.1 × 10^4^±1.2 × 10^4^ 2.6 × 10^4^±0.62 × 10^4^	5.4 × 10^4^±1.6 × 10^4^ 2.9 × 10^4^±0.91 × 10^4^	6.1 × 10^4^±3.1 × 10^4^ 3.1 × 10^4^±0.41 × 10^4^	7.9 × 10^4^±2.1 × 10^4^ 3.5 × 10^4^±1.1 × 10^4^
PA-inactive patients					
miR-122 miR486	3.1 × 10^4^±0.22 × 10^4^ 1.9 × 10^4^±0.81 × 10^4^	4.7 × 10^4^±0.89 × 10^4^ 3.9 × 10^4^±1.2 × 10^4^	6.1 × 10^4^±2.3 × 10^4^ 4.3 × 10^4^±1.3 × 10^4^	7.6 × 10^4^± 1.5 × 10^4^ 5.3 × 10^4^±1.3 × 10^4^	8.6 × 10^4^±2.3 × 10^4^ 6.9 × 10^4^±0.81 × 10^4^

## Discussion

MicroRNAs (miRNAs) are negative gene regulators with more than 2,500 copies in the human genome [21–28] and have been shown to control many crucial biological processes, such as cellular proliferation, cellular differentiation and apoptosis. The location of miRNAs in exosomes provides sufficient stability, thus underscoring their role in diverse cellular processes, including disease pathogenesis and antiviral defense [29, 30]. In addition, an understanding of the pathogenesis of human virus-associated complications is important for developing effective means of preventing and treating liver diseases [65, 66].

Currently, the identification of noninvasive biological biomarkers, such as miRNAs, that can be used to screen high-risk patients for diagnosis is urgently needed [67, 68]. Changes in miRNA expression profiles have been documented in liver diseases, particularly in the context of HCV infection, spanning both the early and advanced stages of liver fibrosis [67, 68]. However, there is limited or no available information regarding the detection of miRNAs and their potential association with physical activity and muscle capacity in individuals with CHC.

Thus, the aim of our biomarker discovery approach was to investigate the potential of using miRNA expression in HCV-infected patients and its correlation with HCV complications in terms of physical activity, pain, and muscle capacity for potential use as noninvasive biomarkers for the early diagnosis of high-risk HCV.

In this study, we initially quantified the serum concentrations of both miR-122 and miR-486 in control subjects and individuals with CHC. Notably, patients with CHC exhibited markedly elevated serum levels of miR-122 and miR-486 in comparison to those in healthy controls. Our findings demonstrated positive correlations between miR-122, miR-486, alanine transaminase (ALT), aspartate transaminase (AST), fibrosis stage, and inflammation activity. However, we did not observe any significant correlations with HCV-RNA levels or viral load. Intriguingly, patients with advanced liver fibrosis (F3-F4) displayed higher expression levels of these measured miRNAs than did those with early liver fibrosis (F0-F1).

Our results align with previous studies reporting of increased circulating levels of miR-122 and miR-486 in patients with hepatitis B virus (HBV) [69–75] and hepatitis C virus (HCV) [74, 75] infections. These elevated levels were found to be significantly associated with liver fibrosis, inflammation scores, and alanine transaminase (ALT) and aspartate transaminase (AST) activity [69–75]. Importantly, no substantial correlation was established between the assessed miRNAs and HCV-RNA levels in human liver biopsies [76–80].

The expression of distinct miRNAs, including serum miR-122, is upregulated in patients with various HCV genotypes, thus suggesting the potential utility of these miRNAs as diagnostic biomarkers for HCV infection [31]. Notably, our study demonstrated correlations between miR-122, miR-486, fibrosis, inflammation, and glucose levels, which could be linked to the progression of hepatic steatosis [75, 81–84].

The release of miRNAs into the serum can occur through passive mechanisms, such as liver cell apoptosis and necrosis, or active processes involving the secretion of exosomes and viral particles. Consequently, the quantification of specific miRNA levels in serum may serve as a significant indicator of ongoing activity within liver cells [83, 84]. Although miRNAs are typically found within exosomes or macrovesicles, they can also exist in the form of free ribonucleoprotein complexes or be transported via the HBV surface antigen (HBsAg) [84–88].

Recently, it was postulated that the presence of miRNAs enriched in muscles in the circulation may be associated with the modulation of metabolic responses induced by physical exercise [89]. Previous studies have demonstrated that the levels of certain miRNAs in skeletal muscle can be altered by physical activity and exercise programs in humans [90] and experimental models [91]. Previous reports have also suggested that to enhance the self-confidence and overall muscle capacity of patients with CHC, improving the physical activity of these patients is a promising option [19, 20]. The bodies of physically active patients with HCV manufacture more ATP, thus indirectly boosting the body’s glutathione levels, which correspondingly improves the resilience of the liver against free radicals and hepatitis C damage [21].

In the current study, we attempted to determine whether there was an association between the expression of miRNAs and both physical activity and muscle capacity in controls and patients with CHC. In patients with CHC with a lower physical activity score, the serum levels of miR-122 and miR-486 were significantly lower than those in control subjects and more physically active patients with CHC. Consistent with our results, miR-486 was demonstrated to be significantly reduced in physically active subjects following focused and regular exercise [92]. However, in those with a sedentary lifestyle or who exhibit lower physical performance, increased levels of miR-486 were reported in the circulation [93, 94].

The decrease in the release of miR-486 from muscle tissue into the bloodstream of physically active individuals may be linked more to the utilization of lipids rather than glucose as the primary energy source during physical activity [92, 93]. This phenomenon could involve the uptake of miR-486 from the blood into muscle tissue. Furthermore, this concept has been supported by previous research that explored the connection between HCV infection and lipid metabolism in the host. Notably, HCV proteins and circulating infectious particles have been associated with and shown to interact with lipid droplets and structures resembling very low-density lipoproteins (VLDLs), which are commonly referred to as lipoviral particles (LVPs). These interactions play a role in facilitating virion assembly and production [93, 94].

In addition, miR-122 was shown to be closely correlated with obesity, as were the levels of lipids, glucose and insulin in patients with a lower physical activity score; this effect correspondingly demonstrated a significant correlation with viral infection. In active patients, lipid utilization and decreased insulin resistance require the uptake of miR-122 from the blood into liver tissues [95, 96]. A wide range of miRNAs were up- or downregulated based upon individual participant, exercise type and the intensity of physical training, thus raising the possibility of manipulating and understanding tissue development, function, and disease progression [97, 98]. Furthermore, it was previously reported that miR-122 is one of the miRNAs that is solely expressed in the muscle, liver and brain [99–101].

In the present study, the levels of LDH, CK, TAC, GSH, and GSSG, as well as the GSH/GSSG ratio, were estimated as potential markers of physical activity and muscle capacity in patients with CHC and control subjects.

In physically active patients with CHC, significantly lower levels of LDH, CK, and GSSG and elevated levels of TAC and GSH were observed, as was a higher GSH/GSSG ratio and a reduction in pain intensity compared to patients with CHC who had lower PA scores (*P* = 0.001) and those in the control group (*p* = 0.01). In addition, miR-122 and miR-486 expression in patients with CHC was positively correlated with LDH and CK levels and negatively correlated with PA scores; TAC, GSH, and GSSG levels; and the GSH/GSSG ratio. In physically active patients with HCV, their bodies manufacture more ATP, thus indirectly boosting the body’s glutathione levels. This correspondingly improves the resilience of the liver against free radicals and hepatitis C damage [21]. Normally, in liver disease, the antioxidant status is significantly reduced as a result of viral infection or hepatotoxic agents [102, 103].

Reports indicate that HCV infection alone or in combination with HBV or HIV is significantly correlated with a reduction in cellular antioxidant mechanisms and elevated oxidative stress, which causes liver cell damage and a decrease in the number of mitochondrial DNA copies [104]. Hence, the cumulative impact of oxidative stress and the depletion of antioxidants may play a crucial role in the progression of liver diseases [105, 106]. Generally, in patients with HCV infection, an increase in GSSG levels and a reduction in TAC and GSH levels and the GSH/GSSG ratio have been reported in response to increased free radical oxidative species [107].

Additionally, physical activity through exercise has demonstrated effectiveness in decreasing inflammation and markers of oxidative stress in individuals with impaired liver function and liver fibrosis, as reported in previous research [86]. Other studies have reported of elevated LDH and CK levels in HCV patients [85–87], and the release of miR-122 and miR-486 was shown to be linked to a noticeable increase in the peak LDH and CK levels in the muscle tissues of infected patients [108, 109]; this miRNA expression was delayed following exercise or physical activity [89–91].

Concerning pain, previous research has indicated a connection between HCV infection and musculoskeletal pain, as well as general pain [92, 93]. In our investigation, we noted a substantial decrease in pain severity among CHC patients who were physically active. Previous studies have shown that physical inactivity is an additional negative parameter affecting chronic pain conditions [94–98], and patients or healthy persons who are regularly physically active may use moderate exercise as a significant tool for the prevention of chronic disease [14, 15].

Moreover, our results suggested that the expression of both miR-122 and miR-468 was negatively correlated with sex among the examined CHC patients, which suggests that miRNA expression could be influenced by sex differences. Previous studies have shown that some miRNAs expressed in certain tissues are sex specific and are significantly more strongly associated with the X chromosome in females than with the Y chromosome [110–113]. Gender differences may be due to inactivation of one of the two X chromosomes occurring in men to maintain balanced expression of the X chromosomes, whereas chromosome inactivation leads to biallelic expression of miRNAs [110–113]. In addition, significant sex differences in miRNA expression have emerged in many patients with liver diseases, including liver cancer and viral hepatitis, and are closely correlated with sex hormones [114, 115]. In addition, our results provided evidence that the expression of miR-122 and miR-468 was significantly associated with an increase in BMI, which is an adiposity marker, in CHC patients with poor physical activity compared to that in patients who are physically active. Previous studies have shown that adipose-derived exosomal miRNAs can regulate gene expression in distant tissues such as the liver and could be considered a novel class of adipokines [116–118]. The importance of circulating miRNAs as novel adipokines in HCV infection remains to be clarified.

Obviously, further studies are necessary to clarify the mechanisms of action and targets of the identified miRNAs miR-122 and miR-486, which are justified noninvasive biomarkers of the pathogenesis of HCV, as well as to correlate their expression levels with poor physical activity, obesity, sex, pain, and muscle fatigue, which are potential prognostic factors for CHC, and which can respond to therapeutic parameters.

## Conclusions

In conclusion, this study demonstrated significant associations between miRNA-122 and miRNA-486 expression levels and various aspects of health in patients with HCV infection and other related viral complications in terms of physical activity, pain perception, adiposity, and muscle fatigue biomarkers. In CHC patients, the expression of cellular miR-122 and miR-486 was shown to play a crucial role in influencing physical activity, pain perception, adiposity, and muscle fatigue biomarkers within this CHC population. Furthermore, elevated levels of miR-122 and miR-486 were associated with increased levels of aspartate aminotransferase (AST), alanine aminotransferase (ALT), lactate dehydrogenase (LDH), and creatine kinase (CK). Additionally, these miRNA expression levels were correlated with fibrosis scores and antioxidant status, thus suggesting their potential utility as biomarkers for disease severity and oxidative stress in patients with HCV infection or its complications. Interestingly, no significant correlation was found between miR-122/miR-486 expression and viral load or HCV RNA expression in the same patients, thus suggesting that these microRNAs may be more closely associated with overall health status and complications of HCV infection than directly impacting viral replication or RNA expression.

Overall, our study provides valuable insights into the potential of miR-122 and miR-486 as noninvasive biomarkers for evaluating the pathogenesis of CHC infection in terms of their correlation with physical activity, pain perception, adiposity, sex, and muscle fatigue in individuals with HCV infection. Further research should explore the mechanistic pathways underlying these associations and their clinical implications for managing HCV-infected patients.

## Data Availability

The datasets used and/or analyzed during the current study are available from the corresponding author on reasonable request.

## References

[1] Westbrook RH, Dusheiko G. Natural history of hepatitis C. J Hepatol. 2014;61(1):S158–68. 10.1016/j.jhep.2014.07.012 25443346

[2] World Health Organization. Initiative for vaccine research (http://www.who.int/vaccine_research/diseases/viral_cancers/en/index2.html).

[3] Thuluvath PJ, Guidinger MK, Fung JJ, Johnson LB, Rayhill SC, Pelletier SJ. Liver transplantation in the United States, 1999–2008. Am J Transpl. 2010;10:1003–19. 10.1111/j.1600-6143.2010.03037.x 20420649

[4] Backus LI, Boothroyd DB, Phillips BR, Belperio P, Halloran J, Mole LA. A sustained virologic response reduces risk of all-cause mortality in patients with hepatitis C. Clin Gastroenterol Hepatol. 2011;9(6):509. e1-516.e1. 21397729 10.1016/j.cgh.2011.03.004

[5] Morgan TR, Ghany MG, Kim HY, et al. Outcome of sustained virological responders with histologically advanced chronic hepatitis C. Hepatology. 2010;52:833–44. 20564351 10.1002/hep.23744 PMC2932862

[6] van der Meer AJ, Veldt BJ, Feld JJ, et al. Association between sustained virological response and all-cause mortality among patients with chronic hepatitis C and advanced hepatic fibrosis. JAMA. 2012;308:2584–93. 23268517 10.1001/jama.2012.144878

[7] Spiegel BM, Younossi ZM, Hays RD, Revicki D, Robbins S, Kanwal F. Impact of hepatitis C on health related quality of life: a systematic review and quantitative assessment. Hepatology. 2005;41:790–800. 15791608 10.1002/hep.20659

[8] Kubo N, Furusyo N, Nakashima H, Kashiwagi K, Hayashi J. Strenuous physical labor is important as a cause of elevated alanine aminotransferase levels in Japanese patients with chronic hepatitis C viremia. Eur J Epidemiol. 2005;20:251–61. 15921043 10.1007/s10654-004-6516-5

[9] El-Zayadi A, Selim O, Hamdy H, El-Tawil A, Badran HM, Attia M, Saeed A. Impact of cigarette smoking on response to interferon therapy in chronic hepatitis C Egyptian patients. World J Gastroenterol. 2004;10:2963–6. 15378774 10.3748/wjg.v10.i20.2963 PMC4576253

[10] Pessione F, Ramond MJ, Njapoum C, Duchatelle V, Degott C, Erlinger S, Rueff B, Valla DC, Degos F. Cigarette smoking and hepatic lesions in patients with chronic hepatitis C. Hepatology. 2001;34:121–5. 11431742 10.1053/jhep.2001.25385

[11] Pol S, Marcellin P. Prise en charge de l’hépatite C en 2008. Gastroenterol Clin Biol. 2008;32:71–3. 10.1016/S0399-8320(08)73268-8 18675183

[12] Thein H, Haber P, Dore G. Quality of life of women living with hepatitis C. J Gastroenterol Hepatol. 2003;18:841–50. 14675259 10.1046/j.1440-1746.2003.03216.x

[13] Dalgard O, Egeland A, Skaug K, Vilimas K, Steen T. Health-related quality of life in active injecting drug users with and without chronic hepatitis C virus infection. Hepatology. 2004;39:74–80. 14752825 10.1002/hep.20014

[14] Roubenoff R, Schmitz H, Bairos L et al. Reduction of abdominal obesity in lipodystrophy associated with human immunodeficiency virus infection by means of diet and exercise: case report and proof of principle CID 2002 ; 34 : 390–3. 10.1086/338402 11774087

[15] Mannerkorpi K, Iversen M. Physical exercise in fibromyalgia and related syndromes Brest. Pract Res Clin Rheumatol. 2003;17:629–47. 10.1016/S1521-6942(03)00038-X 12849716

[16] Haykowsky MJ, Liang Y, Pechter D, Jones LW, McAlister FA, Clark AM. A meta-analysis of the effect of exercise training on left ventricular remodeling in heart failure patients: the benefit depends on the type of training performed. J Am Coll Cardiol. 2007;49:2329–36. 17572248 10.1016/j.jacc.2007.02.055

[17] Harrison SA, Day CP. Benefits of lifestyle modification in NAFLD. Gut. 2007;56:1760–9. 17911352 10.1136/gut.2006.112094 PMC2095707

[18] Sørensen JB, Kragstrup J, Kjaer K, Puggaard L. Exercise on prescription: trial protocol and evaluation of outcomes. BMC Health Serv Res. 2007;7:36. 17331263 10.1186/1472-6963-7-36 PMC1820778

[19] Gapinski MA, Zucker DM. Factors influencing the development of a hepatitis C exercise protocol: a literature review. Gastroenterol Nurs. 2005;28:S10–8. 15976555 10.1097/00001610-200505001-00003

[20] Payen JL, Pillard F, Mascarell V, Rivière D, Couzigou P, Kharlov N. Is physical activity possible and beneficial for patients with hepatitis C receiving pegylated interferon and Ribavirin therapy? Gastroenterol Clin Biol. 2009;33(1 Pt 1):8–14. 19070444 10.1016/j.gcb.2008.10.009

[21] Barbaro G, Di Lorenzo G, Soldini M, et al. Hepatic glutathione deficiency in chronic hepatitis C: quantitative evaluation in patients who are HIV positive and HIV negative and correlations with plasmatic and lymphocytic concentrations and with the activity of the liver disease. Am J Gastroenterol. 1996;91(12):2569–73. 8946988

[22] Zhang JF, Fu WM, He ML, et al. MiRNA-20a promotes osteogenic differentiation of human mesenchymal stem cells by co-regulating BMP signaling. RNA Biol. 2011;8:829–38. 21743293 10.4161/rna.8.5.16043

[23] Wan G, Xie W, Liu Z, et al. Hypoxia-induced MIR155 is a potent autophagy inducer by targeting multiple players in the MTOR pathway. Autophagy. 2014;10:70–9. 24262949 10.4161/auto.26534 PMC4389881

[24] Chekulaeva M, Filipowicz W. Mechanisms of miRNA-mediated post-transcriptional regulation in animal cells. Curr Opin Cell Biol. 2009;21:452–60. 19450959 10.1016/j.ceb.2009.04.009

[25] Valadi H, Ekström K, Bossios A, et al. Exosome-mediated transfer of mRNAs and microRNAs is a novel mechanism of genetic exchange between cells. Nat Cell Biol. 2007;9:654–9. 17486113 10.1038/ncb1596

[26] Mitchell PS, Parkin RK, Kroh EM et al. Circulating microRNAs as stable blood-based markers for cancer detection. Proc. Natl Acad Sci USA. 2008; 105: 10513–10518. 10.1073/pnas.0804549105 PMC2492472 18663219

[27] Mall C, Rocke DM, Durbin-Johnson B, et al. Stability of miRNA Ihuman urine supports its biomarker potential. Biomark Med. 2013;7:623–31. 23905899 10.2217/bmm.13.44 PMC3885156

[28] Zhou Q, Li M, Wang X, et al. Immune-related microRNAs are abundant in breast milk exosomes. Int J Biol Sci. 2012;8:118–23. 22211110 10.7150/ijbs.8.118 PMC3248653

[29] Kozomara A, Griffiths-Jones S, miRBase. Annotating high confidence microRNAs using deep sequencing data. Nucleic Acids Res. 2014;42:D68–73. 24275495 10.1093/nar/gkt1181 PMC3965103

[30] Zhou R, O’Hara SP, Chen XM. MicroRNA regulation of innate immune responses in epithelial cells. Cell Mol Immunol. 2011;8:371–9. 21725335 10.1038/cmi.2011.19 PMC4012889

[31] Shwetha S, Gouthamchandra K, Chandra M, et al. Circulating miRNA profile in HCV infected serum: novel insight into pathogenesis. Sci Rep. 2013;3:1555. 23549102 10.1038/srep01555 PMC3615409

[32] Lagos-Quintana M, Rauhut R, Yalcin A, et al. Identification of tissue-specific microRNAs from mouse. Curr Biol. 2002;12:735–9. 12007417 10.1016/S0960-9822(02)00809-6

[33] Jopling CL, Yi M, Lancaster AM, Lemon SM, Sarnow P. Modulation of hepatitis C virus RNA abundance by a liver-specific MicroRNA. Science. 2005;309:1577–81. 16141076 10.1126/science.1113329

[34] Baggish AL, Hale A, Weiner RB, Lewis GD, Systrom D, Wang F, Wang TJ, Chan S. Dynamic regulation of circulating microRNA during acute exhaustive exercise and sustained aerobic exercise training. J Physiol. 2011;589:3983–94. 21690193 10.1113/jphysiol.2011.213363 PMC3179997

[35] Laterza OF, Lim L, Garrett-Engele PW, et al. Plasma MicroRNAs as sensitive and specific biomarkers of tissue injury. Clin Chem. 2009;55:1977–83. 19745058 10.1373/clinchem.2009.131797

[36] Mitchell PS, Parkin RK, Kroh EM, et al. Circulating microRNAs as stable blood-based markers for cancer detection. Proc Natl Acad Sci U SA. 2008;105:10513–8. 10.1073/pnas.0804549105 PMC2492472 18663219

[37] Banzet S, Chennaoui M, Girard O, Racinais S, Drogou C, Chalabi H, Koulmann N. Changes in circulating microRNAs levels with exercise modality. JApplPhysiol1985. 2013;115(9):1237–44. 10.1152/japplphysiol.00075.2013 23950168

[38] Hoffmann TW, Duverlie G, Bengrine A. MicroRNAs and hepatitis C virus: toward the end of miR-122 supremacy. Virol J. 2012;9:109. 22691570 10.1186/1743-422X-9-109 PMC3489824

[39] Zhao W, Duan X, Li Y, Li S, Liu B, et al. MicroRNA and its potential use for the treatment of Hepatitis C virus infection. J Bioanal Biomed. 2014;6:e125. 10.4172/1948-593X.1000e125

[40] Lu J, Getz G, Miska EA, Alvarez-Saavedra E, Lamb J, Peck D, Sweet-Cordero A, Ebert BL, Mak RH, Ferrando AA, Downing JR, Jacks T, Horvitz HR, Golub TR. MicroRNA expression profiles classify human cancers. Nature. 2005;435:834–8. 15944708 10.1038/nature03702

[41] Poy MN, Eliasson L, Krutzfeldt J, Kuwajima S, Ma X, Macdonald PE, Pfeffer S, Tuschl T, Rajewsky N, Rorsman P, Stoffel M. A pancreatic islet-specific microRNA regulates insulin secretion. Nature. 2004;432:226–30. 15538371 10.1038/nature03076

[42] Abdalla MA, Haj-Ahmad Y. Promising candidate urinary MicroRNA biomarkers for the Early Detection of Hepatocellular Carcinoma among High-Risk Hepatitis C Virus Egyptian patients. J Cancer. 2012;3:19–31. 10.7150/jca.3.19. 22211142 10.7150/jca.3.19 PMC3245605

[43] Liu B, Sun J, Lei X, Zhu Z, Pei C, Qin L. MicroRNA-486-5p suppresses TGF-β2-induced proliferation, invasion and epithelial-mesenchymal transition of lens epithelial cells by targeting Smad2. J Biosci. 2017;42(4):575–84. 10.1007/s12038-017-9709-2. 29229876 10.1007/s12038-017-9709-2

[44] Zhang X, Zhang T, Yang K, Zhang M, Wang K. miR-486- 5p suppresses prostate cancer metastasis by targeting snail and regulating epithelial–mesenchymal transition. Oncotargets Therapy. 2016;9:6909–14. 27877055 10.2147/OTT.S117338 PMC5108614

[45] Guan Z, Tan J, Gao W, Li X, Yang Y, Li X, Li Y, Wang Q. Circular RNA hsa_circ_0016788 regulates hepatocellular carcinoma tumorigenesis through miR-486/CDK4 pathway. J Cell Physiol. 2018;234(1):500–8. 10.1002/jcp.26612. Epub 2018 Jun 19. 29923236 10.1002/jcp.26612

[46] Ji L, Lin Z, Wan Z, Xia S, Jiang S, Cen D, Cai L, Xu J, Cai X. Mir-486-3p mediates hepatocellular carcinoma sorafenib resistance by targeting FGFR4 and EGFR. Cell Death Dis. 2020;11(4):250. 10.1038/s41419-020-2413-4. 32313144 10.1038/s41419-020-2413-4 PMC7170966

[47] Kim J, Lee C, Shin Y, Wang S, Han J, Kim M, Kim JM, Shin SC, Lee BJ, Kim TJ, Jung Y. sEVs from tonsil-derived mesenchymal stromal cells alleviate activation of hepatic stellate cells and liver fibrosis through miR-486-5p. Mol Ther. 2021;29(4):1471–86. 10.1016/j.ymthe.2020.12.025. 33348053 10.1016/j.ymthe.2020.12.025 PMC8058446

[48] Zhang Q, Cao LY, Cheng SJ, Zhang AM, Jin XS, Li Y. P53-induced microRNA-1246 inhibits the cell growth of human hepatocellular carcinoma cells by targeting NFIB. Oncol Rep. 2015;33:1335–41. 25591821 10.3892/or.2015.3715

[49] Abdel-Hamid M, El-Daly M, Molnegren V, et al. Genetic diversity in hepatitis C virus in Egypt and possible association with hepatocellular carcinoma. J Gen Virol. 2007;88:1526–31. 17412982 10.1099/vir.0.82626-0

[50] Allam A, Gabr S, Ajarem J, Abdel-Maksoud M. Bcl-2 and p53 expression in hepatic tissues of Egyptian patients with chronic hepatitis C. J Pak Med Assoc. 2015;65:1186–92. 26564290

[51] Batts KP, Ludwig J. Chronic hepatitis. An update on terminology and reporting. Am J Surg Pathol. 1995;19:1409–17. 7503362 10.1097/00000478-199512000-00007

[52] Gabr SA, Alghadir AH, Allam AA, Ajarem J, Al-Basher G, Abdel-Maksoud MA, Ghfar AA, Aboud A. Correlation between vitamin D levels and apoptosis in geriatric patients infected with hepatitis C virus genotype 4. Clin Interv Aging. 2016;11:523–33. 10.2147/CIA.S104599. 27217734 10.2147/CIA.S104599 PMC4862759

[53] Thorgeirsson SS, Grisham JW. Molecular pathogenesis of human hepatocellular carcinoma. Nat Genet. 2002;31:339–46. 12149612 10.1038/ng0802-339

[54] Murakami Y, Yasuda T, Saigo K, Urashima T, Toyoda H, Okanoue T, Shimotohno K. Comprehensive analysis of microRNA expression patterns in hepatocellular carcinoma and non-tumorous tissues. Oncogene. 2006;25:2537–45. 16331254 10.1038/sj.onc.1209283

[55] Aoi W, Ichikawa H, Mune K, Tanimura Y, Mizushima K, Naito Y, Yoshikawa T. Muscle-enriched microRNA miR-486 decreases in circulation in response to exercise in young men.Front Physiol. 2013 11;4:80. 10.3389/fphys.2013.00080 PMC3622901 23596423

[56] Alghadir AH, Gabr SA, Anwer S, Al-Eisa E. Fatigue and oxidative stress response to physical activity in type 2 diabetic patients. Int J Diabetes Dev Ctries. 2016;36:59–64. 10.1007/s13410-015-0420-2

[57] Al-Eisa ES, Alghadir AH, Gabr SA. Correlation between vitamin D levels and muscle fatigue risk factors based on physical activity in healthy older adults. Clin Interv Aging. 2016;11:513–22. 27217733 10.2147/CIA.S102892 PMC4862760

[58] Hsu MF, Yu SH, Korivi M et al. Hormetic Property of Ginseng Steroids on Anti-Oxidant Status against Exercise Challenge in Rat Skeletal Muscle. Antioxidants (Basel). 2017; 6(2). pii: E36. 10.3390/antiox6020036 PMC5488016 28534811

[59] Bull FC, Maslin TS, Armstrong T. Global physical activity questionnaire (GPAQ): nine country reliability and validity study. J Phys Act Health. 2009;6:790–804. 20101923 10.1123/jpah.6.6.790

[60] Trinh OT, Nguyen ND, van der Ploeg HP, Dibley MJ, Bauman A. Test-retest repeatability and relative validity of the global physical activity questionnaire in a developing country context. J Phys Act Health. 2009;6(Suppl 1):S46–53. 19998849 10.1123/jpah.6.s1.s46

[61] Bijur PE, Silver W, Gallagher EJ. Reliability of the visual analog scale for measurement of acute pain. Acad Emerg Med. 2001;8:1153–7. 11733293 10.1111/j.1553-2712.2001.tb01132.x

[62] Todd KH, Funk KG, Funk JP, Bonacci R. Clinical significance of reported changes in pain severity. Ann Emerg Med. 1996;27:485–9. 8604867 10.1016/S0196-0644(96)70238-X

[63] Zhang Y, Jia Y, Zheng R, Guo Y, Wang Y, et al. Plasma microRNA-122 as a biomarker for viral-, alcohol-, and chemical-related hepatic diseases. Clin Chem. 2010;56:1830–8. 20930130 10.1373/clinchem.2010.147850

[64] Xu J, Wu C, Che X, Wang L, Yu D, et al. Circulating microRNAs, miR-21, miR-122, and miR-223, in patients with hepatocellular carcinoma or chronic hepatitis. Mol Carcinog. 2011;50:136–42. 21229610 10.1002/mc.20712

[65] Bihrer V, Friedrich-Rust M, Kronenberger B, et al. Serum miR-122 as a biomarker of necroinflammation in patients with chronic hepatitis C virus infection. Am J Gastroenterol. 2011;106(9):1663–9. 21606975 10.1038/ajg.2011.161

[66] Pineau P, Volinia S, McJunkin K, et al. miR-221 overexpression contributes to liver tumorigenesis. Proc Natl Acad Sci U S A. 2010;107:264–9. 20018759 10.1073/pnas.0907904107 PMC2806773

[67] Sarasin-Filipowicz M, Krol J, Markiewicz I, Heim MH, Filipowicz W. Decreased levels of microRNA miR-122 in individuals with hepatitis C responding poorly to interferon therapy. Nat Med. 2009;15:31–3. 19122656 10.1038/nm.1902

[68] Morita K, Taketomi A, Shirabe K, et al. Clinical significance and potential of hepatic microRNA-122 expression in hepatitis C. Liver Int. 2011;31:474–84. 21199296 10.1111/j.1478-3231.2010.02433.x

[69] Hayes CN, Chayama K. MicroRNAs as biomarkers for Liver Disease and Hepatocellular Carcinoma. Int J Mol Sci. 2016;17(3):280. 26927063 10.3390/ijms17030280 PMC4813144

[70] Zheng QF, Zhang JY, Wu JS, et al. Upregulation of miRNA-130a represents good prognosis in patients with HBV-Related Acute-on-chronic liver failure: a prospective study. Med (Baltim). 2016;95(6):e2639. 10.1097/MD.0000000000002639 PMC4753881 26871786

[71] Caster PV, Brandenburger T, Strahl T, et al. Circulating microRNA122, 21 and 223 as potential markers of liver injury following warm ischaemia and reperfusion in rats. Mol Med Rep. 2015;12:3146–50. 25954995 10.3892/mmr.2015.3742

[72] Negro F, Sanyal AJ. Hepatitis C virus, steatosis and lipid abnormalities: clinical and pathogenic data. Liver Int. 2009;29(Suppl 2):26–37. 19187070 10.1111/j.1478-3231.2008.01950.x

[73] Sanyal AJ. Role of insulin resistance and hepatic steatosis in the progression of fibrosis and response to treatment in hepatitis C. Liver Int. 2011;31(Suppl 1):23–8. 21205134 10.1111/j.1478-3231.2010.02397.x

[74] Novellino L, Rossi RL, Bonino F, et al. Circulating hepatitis b surface antigen particles carry hepatocellular microRNAs. PLoS ONE. 2012;7:280. 10.1371/journal.pone.0031952 PMC3314627 22470417

[75] Liu AM, Yao TJ, Wang W, et al. Circulating miR-15b and miR-130b in serum as potential markers for detecting hepatocellular carcinoma: a retrospective cohort study. BMJ Open. 2012;2:e000825. 22403344 10.1136/bmjopen-2012-000825 PMC3308260

[76] Turchinovich A, Weiz L, Langheinz A, Burwinkel B. Characterization of extracellular circulating microrna. Nucleic Acids Res. 2011;39:7223–33. 21609964 10.1093/nar/gkr254 PMC3167594

[77] Arroyo JD, Chevillet JR, Kroh EM et al. Argonaute2 complexes carry a population of circulating microRNAs independent of vesicles in human plasma. Proc. Natl. Acad. Sci. USA. 2011;108:5003–5008. 10.1073/pnas.1019055108 PMC3064324 21383194

[78] Gallo A, Tandon M, Alevizos I, Illei GG. The majority of microRNAs detectable in serum and saliva is concentrated in exosomes. PLoS ONE. 2012;7:280. 10.1371/journal.pone.0030679 PMC3302865 22427800

[79] Aoi W, Ichikawa H, Mune K, Tanimura Y, Mizushima K, Naito Y, Yoshikawa T. Muscle-enriched microRNA miR-486 decreases in circulation in response to exercise in young men. Front Physiol. 2013;4:80. 23596423 10.3389/fphys.2013.00080 PMC3622901

[80] Aoi W, Naito Y, Mizushima K, Takanami Y, Kawai Y, Ichikawa H, et al. The microRNA miR-696 regulates PGC-1{alpha} in mouse skeletal muscle in response to physical activity. Am J Physiol Endocrinol Metab. 2010;298:E799–806. 20086200 10.1152/ajpendo.00448.2009

[81] Nielsen S, Scheele C, Yfanti C, et al. Muscle specific microRNAs are regulated by endurance exercise in human skeletal muscle. J Physiol. 2010;588:4029–37. 20724368 10.1113/jphysiol.2010.189860 PMC3000590

[82] Konishi H, Ichikawa D, Komatsu S, et al. Detection of gastric cancer-associated microRNAs on microRNA microarray comparing pre- and post-operative plasma. Br J Cancer. 2012;106:740–7. 22262318 10.1038/bjc.2011.588 PMC3322946

[83] Wang GK, Zhu JQ, Zhang JT, et al. Circulating microRNA: a novel potential biomarker for early diagnosis of acute myocardial infarction in humans. Eur Heart J. 2010;31:659–66. 20159880 10.1093/eurheartj/ehq013

[84] Popescu CI, Dubuisson J. Review role of lipid metabolism in hepatitis C virus assembly and entry. Biol Cell. 2009;102(1):63–74. 19857204 10.1042/BC20090125

[85] André P, Komurian-Pradel F, Deforges S. Characterization of low- and very-low-density hepatitis C virus RNA-containing particles. J Virol. 2002;76(14):6919–28. 12072493 10.1128/JVI.76.14.6919-6928.2002 PMC136313

[86] Chen X, Liang H, Zhang J, Zen K, Zhang CY. Secreted microRNAs: a new form of intercellular communication. Trends Cell Biol. 2012;22:125–32. 22260888 10.1016/j.tcb.2011.12.001

[87] Leuenberger N, Schumacher YO, Pradervand S et al. Circulating microRNAs as biomarkers for detection of autologous blood transfusion. PLoS ONE 2013; 8 Article e66309. 10.1371/journal.pone.0066309 PMC3688786 23840438

[88] Russell AP, Lamon S, Exercise. Skeletal muscle and circulating microRNAs. Prog Mol Biol Transl Sci. 2015;135:471–96. 26477927 10.1016/bs.pmbts.2015.07.018

[89] Lagos-Quintana M, Rauhut R, Yalcin A, Meyer J, Lendeckel W, Tuschl T. Identification of tissue-specific microRNAs from mouse. Curr Biol. 2002;12:735–9. 12007417 10.1016/S0960-9822(02)00809-6

[90] Donaldson A, Natanek SA, Lewis A, Man WD, Hopkinson NS, Polkey MI, Kemp PR. Increased skeletal muscle-specific microRNA in the blood of patients with COPD. Thorax. 2013;68(12):1140–9. 23814167 10.1136/thoraxjnl-2012-203129 PMC3841809

[91] Cheng S-B, Liu H-T, Chen S-Y, et al. Changes of oxidative stress, glutathione, and its dependent antioxidant enzyme activities in patients with Hepatocellular Carcinoma before and after Tumor Resection. PLoS ONE. 2017;12(1):e0170016. 28081247 10.1371/journal.pone.0170016 PMC5231264

[92] Terry L, Sprinz E, Stein R, Medeiros NB, Oliveira J, Ribeiro JP. Exercise training in HIV-1-infected individuals with dyslipidemia and lipodystrophy. Med Sci Sports Exerc. 2006;38:411–7. 16540826 10.1249/01.mss.0000191347.73848.80

[93] Baum MK, Sales S, Jayaweera DT, et al. Coinfection with Hepatitis C virus, oxidative stress and antioxidant status in HIV-positive drug users in Miami. HIV Med. 2011;12(2):78–86. 20500231 10.1111/j.1468-1293.2010.00849.x PMC2974022

[94] de Mendoza C, Sánchez-Conde M, Timmermans E, et al. Mitochondrial DNA depletion in HIV-infected patients is more pronounced with chronic hepatitis C and enhanced following treatment with pegylated interferon plus Ribavirin. Antivir Ther. 2005;10:557–61. 16038482 10.1177/135965350501000410

[95] 17, Stehbens WE. Oxidative stress in viral Hepatitis and AIDS. Exp Mol Path. 2004;77:121–32. 15351235 10.1016/j.yexmp.2004.04.007

[96] Lim HL, Myers BM, Hamilton BA, Davis GL, Lau JY. Plasma glutathione concentration in patients with chronic hepatitis C virus infection. J Viral Hepat. 1995;2(4):211–4. 7489349 10.1111/j.1365-2893.1995.tb00031.x

[97] Oh S, Tanaka K, Warabi E, Shoda J. Exercise reduces inflammation and oxidative stress in obesity-related liver diseases. Med Sci Sports Exerc. 2013;45(12):2214–22. 23698242 10.1249/MSS.0b013e31829afc33

[98] Mastoi AA, Devrajani BR, Shah SZ, World, et al. J Gastroenterol. 2010;16(5):603–7. 10.3748/wjg.v16.i5.603 PMC2816273 20128029

[99] Turk R, Sterrenburg E, de Meijer EJ, et al. Muscle regeneration in dystrophin-deficient mdx mice studied by gene expression profiling. BMC Genomics. 2005;6:98. 16011810 10.1186/1471-2164-6-98 PMC1190170

[100] Bayer PM, Gabl F, Gergely T, h, Zazgornik J. Isoenzymes of creatine kinase in the perinatal period (author’s transl. J Clin Chem Clin. 1977;15:349–52. 894207

[101] Wang YX, Rudnicki MA. Satellite cells, the engines of muscle repair. Nat Rev Mol Cell Biol. 2012;13:127–33. 10.1038/nrm3265 22186952

[102] Järvinen TAH, Järvinen TLN, Kääriäinen M, Kalimo H, Järvinen M. Muscle injuries Biology and Treatment. Am J Sports Med. 2005;33:745–64. 15851777 10.1177/0363546505274714

[103] Lee J, Chang RW, Ehrlich-Jones L, et al. Sedentary behavior and physical function: Objective evidence from the Osteoarthritis Initiative. Arthritis Care Res (Hoboken). 2015;67(3):366–73. 25155652 10.1002/acr.22432 PMC4336845

[104] Sohn MW, Manheim LM, Chang RW, et al. Sedentary behavior and blood pressure control among osteoarthritis initiative participants. Osteoarthritis Cartilage. 2014;22:1234–40. 25042550 10.1016/j.joca.2014.07.007 PMC4159385

[105] Flynn MA, McNeil DA, Maloff B, et al. Reducing obesity and related chronic disease risk in children and youth: a synthesis of evidence with ‘best practice’ recommendations. Obes Rev. 2006;7(Suppl 1):7–66. 16371076 10.1111/j.1467-789X.2006.00242.x

[106] Dunlop D, Song J, Arnston E, et al. Sedentary time in U.S older adults Associated with disability in activities of Daily Living Independent of Physical Activity. J Phys Act Health. 2015;12(1):93–101. 24510000 10.1123/jpah.2013-0311 PMC4153790

[107] Semanik PA, Lee J, Song J, et al. Accelerometer-monitored sedentary behavior and observed physical function loss. Am J Public Health. 2015;105:560–6. 25602883 10.2105/AJPH.2014.302270 PMC4330824

[108] Durstine JL, Gordon B, Wang Z, Luo X. Chronic disease and the link to physical activity. J Sport Health Sci. 2013;2(1):3–11. 10.1016/j.jshs.2012.07.009

[109] Hassett AL, Williams DA. Non-pharmacological treatment of chronic widespread musculoskeletal pain. Best Pract Res Clin Rheumatol. 2011;25:299–309. 22094203 10.1016/j.berh.2011.01.005

[110] Kraczkowska W, Stachowiak L, Pławski A, Jagodzi ´nski PP. Circulating miRNA as potential biomarkers for diabetes mellitus type 2: should we focus on searching for sex differences? J Appl Genet. 2022;63:293–303. 34984663 10.1007/s13353-021-00678-5 PMC8979931

[111] Hasakova K, Bezakova J, Vician M, Reis R, Zeman M, Herichova I. Gender-dependent expression of leading and passenger strand of miR-21 and miR-16 in human colorectal Cancer and adjacent colonic tissues. Physiol Res. 2017;66:S575–82. 29355387 10.33549/physiolres.933808

[112] Bellenghi M, Puglisi R, Pontecorvi G, De Feo A, Carè A, Mattia G. Sex and gender disparities in Melanoma. Cancers. 2020;12:1819. 32645881 10.3390/cancers12071819 PMC7408637

[113] Tomeva E, Krammer UDB, Switzeny OJ, Haslberger AG, Hippe B. Sex-Specific miRNA Differences in Liquid Biopsies from Subjects with Solid Tumors and Healthy Controls. Epigenomes 2023, 7, 2. 10.3390/epigenomes7010002 PMC9844450 36648863

[114] Chen PJ, Yeh SH, Liu WH, Lin CC, Huang HC, Chen CL, Chen DS, Chen PJ. Androgen pathway stimulates microRNA-216a transcription to suppress the tumor suppressor in lung cancer-1 gene in early hepatocarcinogenesis. Hepvbatology. 2012;56:632–43. 10.1002/hep.25695 22392644

[115] Liu WH, Yeh SH, Lu CC, Yu SL, Chen HY, Lin CY, Chen DS, Chen PJ. MicroRNA-18a prevents estrogen receptor-alpha expression, promoting proliferation of hepatocellular carcinoma cells. Gastroenterology. 2009;136:683–93. 19027010 10.1053/j.gastro.2008.10.029

[116] Thomou T, Mori MA, Dreyfuss JM, Konishi M, Sakaguchi M, Wolfrum C, Rao TN, Winnay JN, Garcia-Martin R, Grinspoon SK, et al. Adipose-derived circulating miRNAs regulate gene expression in other tissues. Nature. 2017;542:450–5. 28199304 10.1038/nature21365 PMC5330251

[117] Li M, Marin-Muller C, Bharadwaj U, Chow KH, Yao Q, Chen C, MicroRNAs. Control and loss of control in human physiology and disease. World J Surg. 2009;33:667–84. 19030926 10.1007/s00268-008-9836-x PMC2933043

[118] Vienberg S, Geiger J, Madsen S, Dalgaard LT. MicroRNAs in metabolism. Acta Physiol. 2017;219:346–61. 10.1111/apha.12681 PMC5297868 27009502

